# Academic Outcomes in Bilingual Children With Developmental Language Disorder: A Longitudinal Study

**DOI:** 10.3389/fpsyg.2019.00531

**Published:** 2019-03-11

**Authors:** Eva Aguilar-Mediavilla, Lucía Buil-Legaz, Raül López-Penadés, Victor A. Sanchez-Azanza, Daniel Adrover-Roig

**Affiliations:** Department of Applied Pedagogy and Educational Psychology, University of the Balearic Islands, Palma, Spain

**Keywords:** developmental language disorder, specific language impairment, student grades, academic achievement, phonological awareness, language comprehension, rapid naming

## Abstract

Previous studies have shown that most English-speaking children with language difficulties show academic difficulties during their schooling. The present study aimed to describe the academic achievement of children speaking Spanish and Catalan with developmental language disorder (DLD) during their primary education and to predict their academic outcomes using several processing skills assessed at the beginning of their schooling. To this end, we followed 28 children during their schooling (6–12 years of age). Participants were divided into two groups, one with DLD (*n* = 14) and a control group (*n* = 14) paired by age, gender, socio-economic status (SES), family language (L1), and classroom. All participants were assessed through different processing skills with the Spanish version of the NEPSY at the beginning of their schooling (age 6): attention (visual attention, auditory attention, and response set), phonological awareness, verbal short-term memory (sentence repetition, and narrative memory), access to language (semantic verbal fluency and rapid naming), and language comprehension (comprehension of verbal commands). At the end of primary education, schools reported the official academic marks at the 1st cycle (6–8 years), 2nd cycle (8–10 years) and 3rd cycle (10–12 years). Direct scores of the processing skills and academic results were used for statistical analyses. Results showed that children with DLD had more frequent grade retention, and their academic marks were significantly lower than those of their peers in all the cycles and for all academic subjects with a high language dependency (all except physical education and mathematics). Those subjects with lower language dependence did not show significant differences (physical education and mathematics). Rapid naming accounted for most of the variance of academic outcomes, followed by phonological awareness, and language comprehension when both groups were taken together. Only rapid naming accounted for academic results in the DLD group and phonological awareness did so for the control group. In sum, children with DLD experienced more academic difficulties during their primary education. Those children (with and without DLD) who experienced difficulties not only with rapid naming but also with phonological awareness and oral language comprehension at the beginning of their schooling showed a higher probability of academic failure.

## Introduction

Developmental Language Disorder (DLD; Bishop et al., 2016, 2017) has been previously named SLI (Stark and Tallal, 1981; Leonard, 1998). According to Stark and Tallal (1981), and later to Leonard (1998), diagnostic criteria for children with SLI included normal cognitive functioning (average scores on non-verbal intelligence tests), normal hearing, normal emotional and social development, and no evidence of brain damage or other neurological deficits; however, these children show a clear delay in language development. Nevertheless, during the last years, a high number of clinicians and researchers has noted that SLI was too exclusive and failed to consider children with both language and non-verbal IQ issues. Under the direction of Dorothy Bishop, the CATALISE consortium (Criteria and Terminology Applied to Language Impairments: Synthesizing the Evidence) conducted a Delphi study that led to a change of the diagnostic label, from SLI to DLD. These authors adapted the criteria in order to not exclude children with a low IQ if they did not show an intellectual disability diagnostic (Bishop et al., 2016, 2017). Thus, the DLD diagnostic criterion is defined as a persistent language delay, not resolved at age 5, affecting everyday life communication and/or learning, without a medical condition that could explain it, such as brain injury, genetic conditions or chromosome disorders, hearing loss, autism spectrum disorders or intellectual disability. This disorder can co-occur with other learning and executive difficulties (e.g., low IQ, attentional deficit, or dyslexia). Comorbidity with other difficulties has created controversy in the scientific community because it could increase heterogeneity in these children, including diverse causes under the same diagnostic label (Volkers, 2018). To avoid this controversy in the present study, we decided to use the name of DLD to follow the CATALISE consensus, but the criteria applied were those used for SLI, and, therefore, all children of the present study showed a normal IQ and did not have any other diagnostic, except language difficulties.

Language difficulties experienced by children with DLD do especially affect morphology and syntax, but can also affect their lexicon, pragmatic skills, and phonology (see Leonard, 1998). However, these linguistic profiles vary according to the specificities of a particular language (Leonard, 2014), and, also, among bilingual children (Aguilar-Mediavilla et al., 2017). Bilingual children with DLD manifest their linguistic deficits in both languages: both languages are learned at a much slower rate than in their age-matched monolingual peers (Hakansson et al., 2003). Nevertheless, in general, they do not show worse profiles compared with monolingual children with DLD, although specific patterns of development have been found in bilinguals with respect to monolinguals (Aguilar-Mediavilla et al., 2017).

Considering this, the specific characteristics of children with DLD should also be explored in other languages than English and in bilingual situations, as it is the case of the present study with Spanish–Catalan bilingual children. In previous studies, bilingual Spanish–Catalan children with DLD have shown several linguistic difficulties, such as omissions in function words, errors in inflected morphology, poor syntactic structure (Serra-Raventós, 2002; Serra-Raventós et al., 2002; Aguilar-Mediavilla et al., 2007), omissions of weak syllables and reductions of syllabic shapes (Aguilar-Mediavilla et al., 2002; Aguilar-Mediavilla and Serra-Raventós, 2006). Additionally, errors in verbs, difficulties in lexical access and poor coherence have also been reported (Sanz-Torrent et al., 2008). Regarding the written modality, studies have shown that children with DLD experience reading difficulties, particularly in decoding at the beginning of their schooling, and in comprehension at its end (Aguilar-Mediavilla et al., 2014; Buil-Legaz et al., 2015, 2016b).

However, not only language characteristics must be considered in the study of DLD. Among other variables, several processing skills, that might underlie language difficulties, should also be considered. This is one of the goals of the present study. In this sense, the few existing studies conducted with bilingual Spanish–Catalan children with DLD have shown persistent difficulties in tasks involved in manipulating segments of words (phonological awareness) and in maintaining verbal units active in phonological memory (non-word repetition or sentence repetition), and other abilities, such as access to underlying phonological representations, are affected early and ameliorate during development (rapid naming or verbal fluency; Buil-Legaz et al., 2016a). Other studies in bilingual children with DLD who had Spanish as one of their languages have reported deficits in phonological memory (Girbau and Schwartz, 2008) and when processing auditory and visual information (Pons et al., 2013). Meanwhile, studies exploring processing deficits in monolingual children with DLD are more abundant. These studies have shown that children with DLD have deficits in phonological processing (Goulandris et al., 2000), phonological memory (Montgomery, 2003; Girbau and Schwartz, 2007), rapid automatized naming (Vandewalle et al., 2010), auditory attention (Aguado Alonso et al., 2006; Buiza-Navarrete et al., 2007; Montgomery et al., 2009), executive functioning (Marton, 2008; Henry et al., 2012) and language processing (see Leonard, 1998; Mendoza, 2012 for a review).

As we have stated in its definition, DLD affects children’s everyday life communication, and it might also affect their academic learning. Previous studies have shown that most children with language difficulties have also reading problems (especially in reading comprehension), and these results remain constant in several languages, such as English (Bishop and Adams, 1990; Catts et al., 2002), Dutch (Vandewalle et al., 2012), French (Zourou et al., 2010), Italian (Brizzolara et al., 2006, 2011), Spanish and Catalan (Aguilar-Mediavilla et al., 2014; Buil-Legaz et al., 2015, 2016b). Learning to read is an essential factor in schooling, and difficulties in the acquisition of this ability hinder learning at school (Espin and Deno, 1993), not only in language-related subjects but also in other subjects (Grimm, 2008; Cromley, 2009; Cowan et al., 2010; Duru and Koklu, 2011; Asimina and Aikaterini, 2015). Therefore, children with DLD are at high risk of scholar failure (Young et al., 2002; Conti-Ramsden et al., 2010), that could also lead to difficulties in long-term employment opportunities (Carroll and Dockrell, 2010; Johnson et al., 2010; Durkin et al., 2012b).

Previous studies have informed that children with DLD have more risk of low academic performance, affecting all academic areas (Hall and Tomblin, 1978; Aram and Nation, 1980; King et al., 1982; Beitchman et al., 1996; Snowling et al., 2001; Young et al., 2002; Asimina and Aikaterini, 2015). Nevertheless, not all the children with DLD obtain lower academic achievements. Beitchman et al. (1996) showed that those children with production and comprehension difficulties seem to have more risk of academic failures at the end of schooling, while children with only articulatory difficulties have less risk (Hall and Tomblin, 1978). Also, Snowling et al. (2001) showed better academic achievements in children who had resolved their language difficulties by the age of five, compared with those that maintained their language difficulties, although both groups obtained worse results than the control group. In this sense, it is important to detect, as soon as possible, which children are at higher risk to fail in their schooling to prevent long-term consequences of language difficulties.

Note that few studies have reported early indicators or predictors of academic outcomes in children with DLD. These studies have shown that early oral language and literacy abilities are highly related to academic outcomes in children with DLD. In this sense, Snowling et al. (2001) found that IQ was the major predictor of academic performance, but literacy skills also accounted for a high proportion of variance, especially in groups with language difficulties. Hall and Segarra (2007) and Durkin et al. (2012a) showed that language was associated with academic outcomes in children with language impairment. Also, Hall and Segarra (2007) found that articulation at earlier years could predict academic skills in children with language impairments. Moreover, Dockrell et al. (2011) showed that literacy has the highest correlations with student grades in secondary education. However, in their study, the former authors reported a regression analysis that depicted a more complex model were academic outcomes were predicted by both literacy, language, and non-verbal measures. Finally, Pentimonti et al. (2016) found that pre-literacy abilities (oral-language, print concepts, alphabet knowledge, and phonological awareness) and, to a lesser extent, socio-emotional abilities (pragmatics, cooperation, and other abilities) predicted academic achievement in children with language difficulties. Therefore, the relation between early indicators and later academic performance is still not clear. Besides, other processing abilities that have been related to language, literacy, or academic achievement in children without language difficulties, such as phonological memory or verbal fluency (Alloway, 2009; Alloway and Alloway, 2010; Alloway and Passolunghi, 2011), have not yet been explored in children with DLD.

Most of these processing skills related to language, reading, or academic achievements, such as phonological memory, auditory attention, rapid naming, phonological awareness, and verbal fluency have been found impaired in children with DLD, and, also, in Spanish–Catalan bilingual children with DLD (Gathercole and Baddeley, 1990; Catts et al., 1999; Girbau and Schwartz, 2007; Zourou et al., 2010; Aguilar-Mediavilla et al., 2014; Buil-Legaz et al., 2016a). It has been shown that these processes are predictors of reading outcomes in children with DLD (Catts et al., 1999; de Bree, 2007; Vandewalle et al., 2010; Zourou et al., 2010; Aguilar-Mediavilla et al., 2014; Buil-Legaz et al., 2015) and of school achievements in children with learning difficulties (Kennard et al., 2000). Although the relation between some of these processing skills (such as phonological awareness and rapid automatized naming) and literacy is well established, less is known about the association between other processing skills measures at pre-school years and later academic achievements (Duncan et al., 2007), and, to date, there are no studies about this relation in bilingual Spanish–Catalan children with DLD.

In this vein, all reported previous studies on academic outcomes in children with DLD have been conducted with English speakers, and it is unclear whether the results would hold in other languages and educational systems. In this sense, the fact that literacy explains academic outcomes in children learning English might be related to language opacity. Several studies have shown consistently that the orthographic depth of a language (the degree of correspondence between graphemes and phonemes) affects the time and accuracy in learning to read (for details see Aro, 2004), being more complex as depth increases. Although some studies have shown that orthographic depth does not seem to affect the cognitive skills needed to develop it (Caravolas et al., 2012), others have found that the impact of phonological awareness (the main literacy predictor) on learning to read was modulated by the transparency of the orthography, being it stronger in deep orthographies (Ziegler et al., 2010). Furthermore, other studies have found that rapid automatized naming could have a greater impact in literacy in orthographically transparent languages (Wimmer, 1993; de Jong and van der Leij, 2003; Brizzolara et al., 2006; Di Filippo et al., 2006) and some results have found that rapid automatized naming is the unique predictor of reading in those children with DLD who are learning a transparent language (Vandewalle et al., 2010). Due to these differences, it could be that literacy is more critical for academic outcomes in English academic systems due to its higher difficulty to learn that language. In contrast, this would also involve literacy to have a lower influence on academic achievement in orthographically more transparent languages, such as Spanish and Catalan.

Therefore, other predictors, beyond those related to literacy (such as phonological awareness, rapid naming, and verbal fluency), must be explored in Spanish–Catalan children. In this sense, we will focus on those processing skills that have been considered as clinical markers of language difficulties (i.e., phonological memory; Conti-Ramsden et al., 2001), and/or have been related with academic failures in children with DLD (i.e., comprehension abilities; Beitchman et al., 1996), and/or have been related with academic failures in other populations (i.e., attention and, also, short term memory; Mulder et al., 2010; Rabiner et al., 2016a,b). With respect short-term memory, this shall be considered as a predictor of academic outcomes because it has been related with academic failures in other clinical populations (i.e., premature children; Mulder et al., 2010). Moreover, a poor phonological memory (a type of short-term memory), is considered a clinical marker of DLD independent of IQ or social economic status (Conti-Ramsden et al., 2001). Besides, the relation between attention difficulties and academic failures has been well established in both clinical populations and typical children (Steinmayr et al., 2010; Rabiner et al., 2016b; Aguilar-Mediavilla et al., 2007). Finally, previous studies have found that children with DLD who have comprehension problems showed a higher probability of having academic failures (Beitchman et al., 1996).

Beyond the differences in languages between the present study and previous ones, most of the studies about academic outcomes have been conducted in the United Kingdom, where the participants attended language units during their schooling (Conti-Ramsden et al., 2012). Educational systems seem to be another key factor in the academic success and social integration in children with DLD, as it is the case for other special educational needs (Kalambouka et al., 2007). The Spanish education system promotes an inclusive education, where all the children attend the same schools and classrooms (Spanish Government, 2006). This inclusive education model should not be a handicap for children with difficulties, such as DLD (Acosta Rodríguez et al., 2010). However, a possible disadvantage of inclusive groups may be that DLD goes unnoticed in most cases. This lack of visibility makes difficult its detection and the application of adequate learning aids in an inclusive context (Klingner et al., 1998; Hurt, 2012; Rice, 2016). Also, the lack of visibility makes especially relevant the early detection and the necessity to predict which children are at risk of academic difficulties before they begin their schooling.

Therefore, the present work has two objectives. First, we aimed to describe the academic results of bilingual Catalan–Spanish children with DLD and to compare them with those of their peers. Second, we intended to identify the best predictors of academic results among processing skills that have been previously related with language impairment, reading difficulties and/or academic difficulties, such as attention, access to information, phonological awareness, verbal short-term memory, and language comprehension. Both goals would help to elucidate better how children with DLD might boost their academic achievement from early schooling.

Regarding the first aim and following previous studies in other languages (Hall and Tomblin, 1978; Aram and Nation, 1980; King et al., 1982; Beitchman et al., 1996; Snowling et al., 2001; Young et al., 2002; Asimina and Aikaterini, 2015), we hypothesized that bilingual Spanish–Catalan children with DLD would show lower academic outcomes than their peers in all subjects. We expected that differences in academic outcomes would be larger in those subjects that are more language-dependent, and differences would increase at later grades given that the language and literacy demands needed for academic achievement are higher.

Concerning the second aim and following previous studies (Snowling et al., 2001; Young et al., 2002; Pentimonti et al., 2016), we expected that those processing skills that have been found more related to literacy (e.g., phonological awareness, rapid naming and verbal fluency), especially in orthographically transparent languages (Vandewalle et al., 2010; Aguilar-Mediavilla et al., 2014; Buil-Legaz et al., 2016b), would account for most of the variance in the global academic outcome. Nevertheless, we expect that other processing abilities that have been found delayed in language difficulties and have been previously related in other clinical populations with academic outcomes (e.g., short-term working memory, attention and language comprehension) might add to the model because literacy learning is easier in Spanish and Catalan (orthographically more transparent languages) and could thus have a lower impact on their academic outcomes.

## Materials and Methods

### Participants

Twenty-eight children participated in this longitudinal study. Fourteen of the participants were students with DLD (six females), and 14 were students without any language disorder (six females). At the beginning of the present study, participants were attending the 1st year of primary school, and at the end of the follow-up, they attended the last year of primary school. See Table 1 for sample description.

**Table 1:** Demographic and linguistic data.

	Controls	DLD	Statistic and *p*
*N*	14	14	
Age at identification	*M* = 5.84, *SD* = 0.23	*M* = 5.73, *SD* = 0.23	*t* = 1.17, *p* = 0.250
Age of last grade	*M* = 11.9, *SD* = 0.2	*M* = 12.4, *SD* = 0.7	*t* = -2.18, *p* = 0.038
**Gender**			
Male	8	8	*X*^2^ = 0.00, *p* = 1.00
Female	6	6	
**SES^∗^**			
Low	1	3	*X*^2^ = 0.69, *p* = 0.712
Medium	8	9	
High	1	1	
**Language used at school**			
Catalan	8	10	*X*^2^ = 2.22, *p* = 0.329
Spanish	2	3	
Bilingual	4	1	
**Family language**			
Catalan	6	3	*X*^2^ = 1.52, *p* = 0.465
Spanish	7	10	
Bilingual	1	1	
**Identification phase at 5 years of age**			
Non-verbal-IQ (WPPSI)	*M* = 110, *SD* = 12.2	*M* = 102.1, *SD* = 9.9	*t* = 1.86, *p* = 0.074
Typical score language (PLON-R)	*M* = 55.9, *SD* = 22.6	*M* = 19.8, *SD* = 6.7	*t* = 7.26, *p* < 0.000
**Language at the end of follow-up (12 years)**			
Percentile language production (sentence repetition NEPSY)	*M* = 60.7, *SD* = 16.4	*M* = 29.4, *SD* = 22.4	*t* = 4.2, *p* < 0.000
Percentile language comprehension (CEG)	*M* = 64.3, *SD* = 24.5	*M* = 40.7, *SD* = 24.5	*t* = 2.56, *p* = 0.016


*M, mean; SD, standard deviation; SES, socio-economic status; PLON-R, Navarra Oral Language Test-Revised; WPPSI, Wechsler Preschool and Primary Scale of Intelligence; SR, Sentence repetition; NEPSY, Developmental Neuropsychological Assessment; CEG, Test of comprehension of grammatical structures.*

*^∗^Four lost data in controls and one in DL*.

#### Identification Phase

In a first step, all schools in Mallorca (Spain) sent us the profiles of every child with language delays (*n* = 85) at the end of the last grade of kindergarten (5 years old). Our research team selected those children whose profile was compatible with a diagnostic of DLD (criteria used were those for SLI; Leonard, 1998). Moreover, children who presented only articulatory problems, communication difficulties and/or being newly arrived from a non-Catalan speaking community, were excluded from the sample (*n* = 23) to avoid the inclusion of sequential second language learners. In a second step, our team evaluated each child selected previously. Language profiles (phonology, morphosyntax, lexicon, and pragmatics) were assessed using the standardized test PLON-R: Navarra Oral Language Test-Revised (*PLON-R: Prueba del Lenguaje Oral de Navarra Revisada*; Cronbach’s α = 0.76 and standard error of measurement = 2; Aguinaga et al., 2004). Non-verbal IQ was measured using the Wechsler Preschool and Primary Scale of Intelligence (WPPSI; Wechsler and de la Cruz, 2001). The speech therapists of the schools reported the history of neurological, social, and emotional difficulties through an open question and reported on the socio-economic status of each child by means of a three levels Likert item (see Table 1) in a written questionnaire elaborated by our team. We also requested their audition records to the Balearic Department of Health. This institution conducted an Otoacoustic Emissions analysis for all children and an audiometric test to those children who failed the Otoacoustic Emissions.

Therefore, for the initial sample (*n* = 20), we only selected those children who had language problems (all of them showed morpho-syntactic difficulties) according to the PLON-R (which considers as language delay those scores from one standard deviation below the mean), with an average non-verbal IQ (more than 85) and with no history of cognitive, auditory, social and neurological damage (Stark and Tallal, 1981; Leonard, 1998). During the follow-up, six participants dropped from the study, and, finally, only 14 children could be included (see Table 1).

Every child with DLD was paired with a control child (*n* = 14) of the same age, sharing the same classroom, and having the maximum number of similarities concerning gender, SES, and family language (either Catalan or Spanish). Language, intelligence, auditory, social, emotional, and neurological variables were also recorded for the control group (see Table 1). None of the demographic variables at this phase showed differences between groups, while all the linguistic proficiency measures showed significant differences. At the identification phase, all children (DLD and controls) had already completed the two previous years of kindergarten, as children had begun school at the age of 3 years.

#### Oral Language at the End of Follow-Up

In order to control for the maintenance of language difficulties in children with DLD and their absence in children on the control group, at the end of the follow-up (12 years of age), oral language was assessed again in both groups (see Table 1). The test PLON-R could not be used again because the oldest age of application is at 6 years. Due to the lack of specific measures at this age, we decided to use two separate measures of language comprehension and language production. Language comprehension was assessed through the CEG (*Test de comprensión de estructuras gramaticales*; Grammatical structures comprehension test; Mendoza-Lara et al., 2005) and language production was evaluated through the sentence repetition subtest of the NEPSY (Developmental Neuropsychological Assessment; Korkman et al., 1998; Aguilar-Alonso et al., 2014). Both, the CEG and the sentence repetition test are considered valid indicators of morpho-syntax and are sensitive for the identification of children with DLD (Conti-Ramsden et al., 2001; Stokes et al., 2006; Leclercq et al., 2014; Muñoz et al., 2014). Results showed significant differences at 12 years between both groups in language comprehension and production (see Table 1).

#### Language of Participants and Language of Assessment

This longitudinal study was conducted in a bilingual Catalan–Spanish context (see Melià and Vanrell, 2017 for a description of language use). Almost all native children in this context are Spanish–Catalan, can be considered as simultaneous bilinguals and experience a similar amount of exposure to both languages. For instance, schooling begins at 3 years of age, and the language used at school is mainly Catalan. Catalan is also a language used in children’s usual contexts. In addition, Spanish is a language that is present in most of the contexts in which children are involved or exposed to (e.g., TV, books); thereby all children learn both languages at a very early age (before 3 years). Therefore, our participants can be all considered as simultaneous Spanish–Catalan bilinguals.

Both languages, Catalan and Spanish, have a high degree of similarity, as they share many words in common (76% of cognates; Lewis et al., 2014), both are orthographically transparent languages and have a similar morphosyntactic structure (for a brief revision see Appendix in Aguilar-Mediavilla et al., 2007). The common bilingual context and the similarity between both languages leads adults to switch from one language to the other during a conversation interchangeably. In very small children, it is also very frequent to observe language mixing even when talking to a single person and/or in the same communicative setting (Aguilar-Mediavilla et al., in press). For example, it is common in this particular dual-language context that an adult asks something in Catalan and the children answers in Spanish or vice versa.

Beyond bilingualism, schools informed about the main language used by the children at home (familiar language: Spanish, Catalan, or both), and the main language used at school (school language: Spanish, Catalan, or both) by means of a written questionnaire. As we have previously stated, each child with DLD was paired with a control child of the same class, age, gender, SES, and familiar language. However, finding a control child who fulfilled all these requirements was not always possible. Nevertheless, as can be seen in Table 1, there were no significant differences between groups in neither the language used at school nor in the family context.

With respect to the language of assessment, the linguistic profile of each child was obtained in the family language by means of the PLON-R (Aguinaga et al., 2004) at the beginning of the study. The NEPSY was administered mainly in Spanish. However, for the subtests that needed a verbal answer, we allowed the children to choose the language of response, and code-switching was also allowed. We also accepted as correct those answers provided in both languages.

### Materials

To assess several processing skills, we selected ten tasks from the Spanish adaptation of the Developmental Neuropsychological Assessment (NEPSY; Korkman et al., 1998) by Aguilar-Alonso et al. (2014). We selected this test because it was adapted to Spanish, also for its good reliability and validity values and because it can be used in the school setting. We believe this is positive in terms of the clinical implications and transference possibilities. In this test, the reliability coefficients were calculated with the Cronbach’s α, the generalizability coefficient or the test–retest stability. We selected those tasks assessing different processing skills that have been previously found delayed in children with DLD and/or have previously been related to language, literacy or academic difficulties in other languages and/or in distinct clinical samples (Gathercole and Baddeley, 1990; Catts et al., 1999; Manis et al., 1999; Snowling et al., 2001; Girbau and Schwartz, 2007; Zourou et al., 2010; Aguilar-Mediavilla et al., 2014; Buil-Legaz et al., 2016a) in order to know which variables could explain a significant proportion of variance of academic outcomes in a Spanish–Catalan bilingual context. The selected tasks measured attention (visual attention [generalizability coefficient = 0.71], auditory attention [test–retest stability coefficient = 0.81], and response set subtasks [test–retest stability coefficient = 0.81]), phonological awareness (phonological awareness subtask [Cronbach’s α = 0.91]), verbal short-term memory (sentence repetition [Cronbach’s α = 0.81], and narrative memory subtasks [test–retest stability coefficient = 0.77]), access to verbal information (semantic verbal fluency [test–retest stability coefficient = 0.74] and rapid naming subtasks [generalizability coefficient = 0.74]), and language comprehension (comprehension of instructions [Cronbach’s α = 0.73]).

Concerning the assessment of attention, we selected three tasks: two for the auditory and one for the visual domain. The first task assessed only auditive attention. In this task, children listened to 180 different words (e.g., “*Negro* [black], *casa* [house], *pronto* [soon], *ROJO* [RED], *círculo* [circle]…”) and when they listened the word “*ROJO*” ([RED]), they were instructed to put a red square in a box. The score was calculated by subtracting the commission errors (maximum score = 180) from the total score (maximum = 60). The second auditory task was the response set task. This is a similar but more complex task since it assesses complex auditory attention, but also working memory and inhibition. The children listened to a different set of 180 words (e.g., “*Allí* [there], *ahora* [now], *AMARILLO* [YELLOW], *fino* [thin], *aburrido* [boring], *AZUL* [BLUE]…”). Moreover, when they listened to the word “*ROJO*” ([RED]), they were instructed to put a yellow square in a box, when they heard the word “*AMARILLO*” ([Yellow]) they had to put a red square, and when they heard “*AZUL*” ([Blue]) children had to put a blue square in the box. The score was calculated by subtracting the commission errors (maxim score 180) from the total score (maximum 72). To assess visual attention, we used an attention to faces task in which children were instructed to encircle all faces that matched two target ones on an A3 page with 96 different faces.

In order to evaluate Phonological awareness, we used a phonological processing task that did not load working memory. In this task, the examiner first showed a picture (total = 14), with three elements on it and named them (e.g., ‘*cocina*’ [kitchen], ‘*niños*’ [children], ‘*gallina’* [hen]). After, the experimenter said only a part of the word by omitting the initial syllable, the final syllable, or some of the phonemes of the word, and the child was instructed to point to its correct drawing. The goal of this task was to assess phonological awareness and phonological knowledge that leads to word recognition.

For verbal short-term memory, an ability related to academic outcomes (Mulder et al., 2010), the two selected measures (sentence repetition, and narrative memory) varied in their sublexical demands. Specifically, in the sentence repetition task, children had to repeat 17 sentences of increasing loadings on phonological memory and grammatical complexity. The first sentences were shorter than the last ones (e.g., sentence: 1. *Duerme bien* [sleep well]; and sentence 17. *El próximo miércoles a las dos de la tarde nuestro equipo de fútbol jugará un partido en un campeonato que se celebrará en el estadio* [Next Wednesday at two o’clock our football team will play a match of a championship to be held at the stadium]). In order to measure a general verbal short-term memory, a narrative memory task was selected. In this task, children must listen to a story and then retell it (e.g., *Juan era un niño cuyo mejor amigo era Sultán* … John was a child whose better friend was Sultán….). If some details were not referred, an induced clue was prompted through questions like *What is the name of the child?*. The child must remember 17 items, that were scored with 2 points in free recall, with 1 point in induced recall and with 0 points when the item was not recalled. The maxim possible score was 34.

Regarding the access to verbal information, we used two different tasks that have been related with reading accuracy of children with DLD in transparent languages (Manis et al., 1999; Aguilar-Mediavilla et al., 2014; Buil-Legaz et al., 2015). First, we assessed the activation and integration of semantic information and articulation by measuring the ability for rapidly and accurately naming the size, color and shape of 20 objects (rapid naming). Children had to provide all correct attributes of each item (e.g., little yellow square; big red circle…) in 1 min of time, and the maximum score was 60 points. Unlike typical rapid automatized tasks, the rapid naming task used here requires accessing to three dimensions instead of one. This could result in an increase of demands in terms of attention and working memory, as compared to typical procedures. Nevertheless, the naming task used here is appropriate to measure lexical access since it preserves the core features of conventional rapid automatized naming tasks such as the elicitation of naming by continuously presented visual stimuli under time pressure (Korkman et al., 1998). Second, we used a semantic verbal fluency task, which involved producing as many words as possible in 1 min of time for two semantic categories (animals and food/drinks). The total number of different words produced was considered as the final score.

Finally, we selected a task that measured oral language comprehension, an ability that has been related with reading comprehension (Buil-Legaz et al., 2016b), and has been considered as a key factor related to the academic failure in children with DLD (Beitchman et al., 1996). In this task, children had to point to one image of a picture that corresponded to the given instruction. Instructions were growing in difficulty from “Show me a big rabbit” to “Show me a cross, the black circle and the red cross.” The maximum possible score was 28.

Scoring for each task was calculated following instructions in the NEPSY manual. A summary of the tasks and an example of each one can be consulted in Appendix A.

Regarding the academic achievements, the schools referred us the official student grades at the end of the primary education period. The official academic report (GESTIB academic report; Conselleria d’Educació del Govern Balear, 2017) includes the grade, which ranges from fail (D or C-; less than 5), pass (C+; between 5 and 6), good (B; between 6 and 7), very good (A-; between 7 and 9), to excellent (A+; 9 or higher than 9). The subjects evaluated were science and social knowledge, artistic education, physical education, Spanish language, Catalan language, English language, and mathematics. The official reports inform grades every 2 years, and not at the end of every course. Therefore, grades were reported at three scholar cycles, namely first (1st and 2nd grades), second (3rd and 4th grades) and third cycle (5th and 6th grades). The report also included information about curriculum adaptations and grade retention.

### Procedure

A group of trained undergraduate students of advanced academic courses and graduates in Psychology administered all tasks of the NEPSY at the children’s schools at the beginning of the first course of primary education, at the age of six. The examiners were also Spanish–Catalan bilinguals and did not know whether the participants belonged to the study group or the control group. Every examiner assessed a child with DLD and his/her paired control, to minimize differences between examiners. All the tasks began with an explanation, followed by one or two example items, to make sure that the child had fully understood the task. Task testing was videotaped with a SONY FS100 digital camera and an electret condenser microphone (sensitivity: -65 ± 3 db) to be subsequently scored by an experienced researcher.

The student grades were transformed into ordinal numbers (five levels; from 1, fail, to 5, excellent), and different arithmetic means were calculated: the average mark of every cycle (average mark by cycles: 1st Cycle, 2nd Cycle, and 3rd Cycle), the average mark of every academic subject (average marks by subjects: science and social knowledge, artistic education, physical education, Spanish language, Catalan language, English language, and mathematics), and a total average mark for all the academic subjects and cycles (average mark). One of the schools did not use grades for the first cycle, and for one child in each group, the average mark has been calculated with only the grades from the second and third cycles. Also, one child in the DLD group left the school the last year of primary education and the average mark was calculated with only the data of the 1st and 2nd cycles.

All the data were analyzed with SPSS 25. To control for potential socio-demographic and linguistic confounding variables between groups, several *X*^2^ tests, and independent group contrasts were applied. For group analyses, mixed ANOVA, and Mann–Whitney’s *U* test were applied, and effect sizes were reported with $\eta_{p}^{2}$ and *r*, respectively. Mixed ANOVA was conducted when assumptions were met (i.e., normality, homogeneity of variances, sphericity; Fouladi and Shieh, 2004) and Mann–Whitney’s *U* test when assumptions for the ANOVA were violated. Moreover, effect sizes were interpreted using Cohen’s (1988) categories for both $\eta_{p}^{2}$ and *r*: small ($\eta_{p}^{2}$ > 0.01; *r* > 0.1), medium ($\eta_{p}^{2}$> 0.06; *r* > 0.3) and large ($\eta_{p}^{2}$ > 0.13; *r* > 0.5) effect size.

Bivariate Pearson correlations and stepwise regression analyses were also applied. We used a stepwise regression analysis to identify which were the best predictors of the academic results among the processing skills assessed. Stepwise regression is the technique of choice when assessing which are the most predictive variables for a given output, reducing the final predictors to a minimum. In each step of this regression method, the variable accounting for the most proportion of variance is introduced in the model, thus reducing the number of variables in the final model. We checked the assumptions for the regression analysis (linear relationship between the dependent and independent variables, multicollinearity, and the quality of residuals) and, overall, results evidenced that the assumptions required to estimate the results reported were met.

The research ethics committee (CER^1^) of the University of the Balearic Islands approved the study and provided full consent. All parents signed a written informed consent at the beginning of each phase of the study.

## Results

### Academic Results

As Table 1 shows, although at the beginning of the schooling the age of both groups was similar, the age of children when completing primary education was higher for the DLD group than for the control group, *t*(26) = -2.18, *p* = 0.038. This difference in the age of both groups at the end of the primary school can be attributed to the fact that children in the DLD group experienced grade retention more frequently than children in the control group (one child in the control group in contrast to the eight in the DLD group; *X*^2^ = 8.02, *p* = 0.005). Moreover, children in the DLD group failed grades earlier (being the second grade the most frequently failed with five children, followed by the fourth grade with two children and the third grade with one child) than in the control group (one child in sixth grade).

Concerning the special academic measures adopted by the schools, only four children with DLD, 29% of the total, had curriculum adaptations. In the control group, no participant had curriculum adaptations, *X*^2^ = 4.7, *p* = 0.031. Regarding the relation between repeating the grade and having curriculum adaptations, a significant association was found in the use of both measures in the children with DLD (*X*^2^ = 4.2, *p* = 0.040).

**FIGURE 1:**
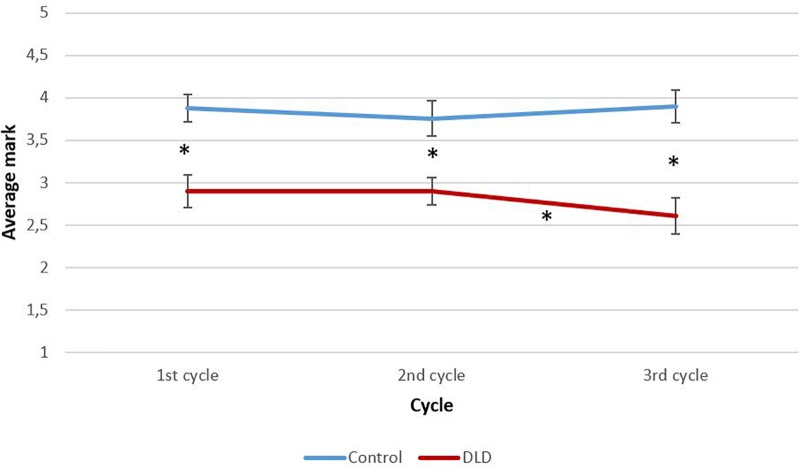
Mean of academic qualification in each cycle (cycle score) of primary education. ^∗^*p* < 0.05.

In order to examine whether children with DLD showed lower academic outcomes than their control peers across cycles, a mixed ANOVA with Group (Control, DLD) as the between-subjects factor, and Cycle (1st, 2nd, and 3rd) as the within-subjects factor was performed on the average marks by cycle (see Table 2 and Figure 1). The main effect of Group was significant, *F*(1,23) = 13.83, *p* = 0.001, $\eta_{p}^{2}$ = 0.376, indicating lower academic outcomes for the DLD group as compared to the control group. The within-subjects effect of Cycle was non-significant, *F*(2,22) = 2.10, *p* = 0.134, $\eta_{p}^{2}$ = 0.084, but the interaction between Group and Cycle yielded significance, *F*(2,22) = 3.32, *p* = 0.045, $\eta_{p}^{2}$ = 0.126. *Post hoc* tests revealed that only the DLD group showed a decrease in the average marks between the 2nd cycle and the 3rd cycle, *p* = 0.049, thus children with DLD showed a decrease in their average cycle results at the end of primary school. All the reported $\eta_{p}^{2}$ values indicate large effect sizes.

**Table 2:** Means and standard deviations (in parenthesis) of academic results for each group.

	Controls				DLD			
	1st cycle	2nd cycle	3rd cycle	Mean	1st cycle	2nd cycle	3rd cycle	Mean
Science and Social	4.0(0.70)	3.9(0.91)	4.0(0.88)	4.0(0.75)	2.7(0.75)	2.9(0.99)	2.3(1.2)	2.6(0.93)
Art education	3.9(0.86)	3.8(0.86)	4.1(0.83)	4.0(0.71)	3.2(1.01)	3.0(0.96)	3.2(0.83)	3.1(0.78)
Physical education	3.9(0.95)	3.9(0.73)	4.36(0.63)	4.1(0.63)	3.5(0.77)	3.6(0.64)	3.5(1.19)	3.5(0.64)
Spanish language	3.7(0.75)	3.6(1.0)	3.9(0.95)	3.7(0.78)	2.7(1.03)	2.6(0.84)	2.3(0.85)	2.5(0.74)
Catalan language	3.46(1.1)	3.7(0.91)	3.6(1.0)	3.6(0.93)	2.5(0.96)	2.86(0.94)	2.2(0.92)	2.4(0.71)
English language	4.2(0.83)	3.8(0.94)	3.9(1.10)	4.0(0.81)	2.9(1.03)	2.64(1.00)	2.0(0.91)	2.5(0.76)
Mathematics	3.9(0.86)	3.4(1.01)	3.6(1.28)	3.65(0.92)	2.9(1.03)	2.86(0.95)	2.7(1.03)	2.8(0.70)
Mean	3.9(0.59)	3.8(0.77)	3.9(0.74)	3.86(0.65)	2.9(0.68)	2.9(0.68)	2.6(0.76)	2.79(0.60)


**FIGURE 2:**
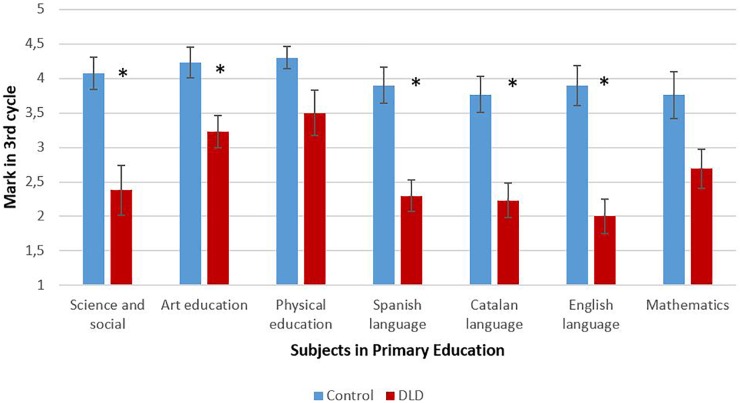
Mean academic results in each subject in the third cycle when completing primary education (3rd cycle score). ^∗^*p* < 0.05.

Moreover, additional analyses on the average marks were conducted in order to disentangle whether these overall average differences in the marks of the 3rd cycle relied on the language dependency of the subjects. Since the distributions of the marks of all specific subjects revealed to be non-normal in the 3rd cycle (Kolmogorov–Smirnov *p*s < 0.045), we opted to analyze the data by means of the Mann–Whitney’s *U* test (see Table 1 and Figure 2). Results showed that children with DLD showed lower average marks than the control group at the end of primary education (3rd cycle score) in all academic subjects (science and social knowledge, *U* = 29.5, *Z* = -3.12, *p* = 0.002, *r* = 0.6; arts education, *U* = 41.5, *Z* = -2.53, *p* = 0.014, *r* = 0.49; Spanish language, *U* = 20, *Z* = -3.45, *p* < 0.0001, *r* = 0.66; Catalan language, *U* = 29.5, *Z* = -3.09, *p* = 0.002, *r* = 0.6; and English language, *U* = 20, *Z* = -3.52, *p* < 0.0001, *r* = 0.68) except for physical education, *U* = 56, *Z* = -1.82, *p* = 0.094, *r* = 0.35, and mathematics, *U* = 53.5, *Z* = -1.87, *p* = 0.068, *r* = 0.36. All the *r*-values in significant differences indicated medium to large effect sizes.

### Correlation and Regression Results: Explaining Academic Outcomes of Primary Education From Pre-school Processing Skills

Pearson correlations and stepwise regression analyses were conducted in order to associate and predict the academic performance from the processing skills measured in earlier years. The average mark represents a global measure of all marks obtained during primary education, and the education government considers it as a comprehensive academic measure at this education level; for this reason, and to reflect the complete academic trajectory of all children, it was selected as the dependent variable. The independent variables introduced were the NEPSY measures collected at the beginning of the 1st year of primary education, at age 6 (visual attention, auditory attention, response set, phonological awareness, sentence repetition, narrative memory, semantic verbal fluency, rapid naming, and comprehension of instructions).

Results showed that all the NEPSY variables measured at 6 years of age, except for semantic verbal fluency, and attention measures (visual attention and response set and auditory attention), correlated with the average mark when both groups were considered together (see Table 3). However, when groups were taken separately, a different set of results was obtained. For the control group, only phonological awareness, visual attention, and comprehension of instructions correlated with academic outcomes. In the DLD group, only rapid naming correlated with the average mark.

**Table 3:** Descriptive statistics and Pearson correlations between academic results in primary education (average mark) and processing skills for both groups, and for control and DLD groups, separately.

Both groups	1	2	3	4	5	6	7	8	9	10
(1) Average mark	1									
(2) Phonological awareness	0.387^∗^	1								
(3) Auditory attention	0.377	0.180	1							
(4) Response set	0.558^∗∗^	0.383	0.718^∗∗^	1						
(5) Rapid naming	0.683^∗∗^	-0.128	0.280	0.344	1					
(6) Visual attention	0.180	0.367	0.168	-0.054	0.072	1				
(7) Comprehension of instructions	0.620^∗∗^	0.093	0.338	0.569^∗∗^	0.411^∗^	0.112	1			
(8) Sentence repetition	0.564^∗∗^	0.357	0.614^∗∗^	0.641^∗∗^	0.258	0.076	0.488^∗∗^	1		
(9) Semantic verbal fluency	0.349	0.162	0.183	0.288	0.427^∗^	-0.060	0.015	0.416^∗^	1	
(10) Narrative memory	0.385^∗^	-0.069	0.425^∗^	0.276	0.327	0.242	0.372	0.595^∗∗^	0.402^∗^	1
*M*	3.33	11.32	34.2	34.48	55.39	6.5	19.57	15.85	20.78	14.18
*SD*	0.82	2.03	22.6	23.47	6.16	11.02	3.28	4.44	7.16	5.67
Skewness	0.12	-0.98	-0.74	-0.15	-1.76	-2.45	-0.31	0.22	0.87	0.29
Kurtosis	-1.19	0.50	-0.79	-1.14	2.71	8.49	1.44	-0.17	1.01	-0.54
**Control group**										
(1) Average mark	1									
(2) Phonological awareness	0.767^∗∗^	1								
(3) Auditory attention	0.245	0.608^∗^	1							
(4) Response set	0.484	0.624^∗^	0.656^∗^	1						
(5) Rapid naming	0.487	0.446	0.204	0.502	1					
(6) Visual attention	0.583^∗^	0.321	0.153	0.310	0.224	1				
(7) Comprehension of instructions	0.544^∗^	0.485	0.360	0.745^∗∗^	0.348	0.330	1			
(8) Sentence repetition	0.480	0.594^∗^	0.380	0.578^∗^	0.416	0.356	0.423	1		
(9) Semantic verbal fluency	0.027	0.145	0.054	0.026	0.421	-0.101	-0.193	0.438	1	
(10) Narrative memory	0.264	0.062	-0.161	0.127	0.190	0.541^∗^	0.290	0.350	0.279	1
*M*	3.86	11.78	48.75	45.25	58.57	8.64	21.21	18.71	23.07	17.07
*SD*	0.65	1.57	8.67	22.21	1.82	5.61	2.57	3.87	7.14	5.63
Skewness	-0.51	-0.69	-0.61	-1.11	-1.46	-0.65	1.17	-0.08	0.86	-0.31
Kurtosis	-0.36	-0.56	-0.94	0.96	1.77	0.09	3.13	-0.24	1.01	-0.13
**DLD group**										
(1) Average mark	1									
(2) Phonological awareness	0.015	1								
(3) Auditory attention	-0.165	-0.065	1							
(4) Response set	0.290	0.147	-0.714^∗∗^	1						
(5) Rapid naming	0.669^∗∗^	-0.447	-0.054	0.109	1					
(6) Visual attention	-0.138	0.351	0.057	-0.338	-0.064	1				
(7) Comprehension of instructions	0.353	-0.309	-0.068	0.153	0.190	-0.087	1			
(8) Sentence repetition	-0.129	0.033	0.449	0.435	-0.367	-0.311	0.050	1		
(9) Semantic verbal fluency	0.379	0.063	0.004	0.328	0.369	-0.170	-0.181	0.071	1	
(10) Narrative memory	-0.233	-0.521	0.349	-0.040	0.066	0.016	-0.004	0.475	0.308	1
*M*	2.79	10.85	23.49	24.53	52.21	14.53	17.92	13.0	18.5	11.28
*SD*	0.60	2.38	34.20	20.65	7.33	6.50	3.14	2.90	6.66	5.67
Skewness	0.74	-0.80	0.29	0.49	-0.90	-1.93	-0.82	-0.70	1.21	0.34
Kurtosis	-0.39	-0.16	-1.23	-0.39	0.04	4.62	-0.71	-0.58	3.05	0.06


*^∗^p < 0.05, ^∗∗^p < 0.001; M, mean; SD, standard deviation.*

Taking both groups together, the stepwise regression analysis performed on the average mark (see Table 4) showed that rapid naming was the variable that explained the most variance (41% variance explained). Adding phonological awareness, the variance explained increased to 64.1%. The final model added comprehension of instructions to rapid naming and phonological awareness and explained 75.8% of the variance. When the stepwise regression analyses were carried out separately for each group, the results were slightly different. For the control group, phonological awareness was the unique predictor of the average mark and accounted for 53.6% of its variance. For the DLD group, rapid naming raised as the unique significant predictor of the average mark, accounting for 37.4% of its variance.

**Table 4:** Statistics from the stepwise regression model, with the mean academic results (average mark) of primary education (from 6 to 12 years) as a predicted variable, and processing skills measures collected at age 6 as independent variables.

Dependent: average mark			
	**Both groups together**			
**Predictor**	**β**	**Adj.*R*^2^**	***F* or *t***	***p***
**Step 1**		0.410	17.65	<0.000
Rapid naming	0.659		4.2	<0.000
**Step 2**		0.641	22.4	<0.000
Rapid naming	0.739		6	<0.000
Phonological awareness	0.493		4	0.001
**Step 3**		0.758	26.01	<0.000
Rapid naming	0.611		5.6	<0.000
Phonological awareness	0.439		4.2	<0.000
Comprehension of instructions	0.367		3.4	0.003
	**Control group**			
**Predictor**	**β**	**Adj.*R*^2^**	***F* or *t***	***p***
**Step 1**		0.536	13.71	0.004
Phonological awareness	0.760		3.7	0.004
	**DLD group**			
**Predictor**	**β**	**Adj.*R*^2^**	***F* or *t***	***p***
**Step 1**		0.374	8.16	0.016
Rapid naming	0.653		2.85	0.016


## Discussion

The aim of this study was twofold. First, we aimed to describe the academic performance of Spanish–Catalan children with DLD in primary school, from 6 to 12 years of age. The second objective was to analyze which early processing skills are related to their academic results and thus, to predict academic outcomes from the beginning of schooling.

Regarding the first objective, the first hypothesis was confirmed, as results showed that children with DLD experienced more academic difficulties during their primary education than their peers without language difficulties. These children failed grades more frequently and earlier than their peers did. Our results showed that the percentage of grade repetition in children with DLD is 25%, slightly higher than the mean percentage of grade repetition in Spain, which is around 30–34% (Instituto Nacional de Evaluación Educativa, 2013; OCDE, 2016). Besides, the academic results of the children with DLD were lower than those of their peers during the three cycles, getting worse in the last cycle of primary education, when language and literacy become more complex. These academic difficulties were evident in high language-related subjects. Furthermore, the effect sizes corresponding to the differences between groups increased as subjects were more language-dependent (large effect sizes in languages; medium effect sizes in science and society; lower effect sizes in art education; and no differences in physical education and mathematics), showing that language difficulties impact particularly subjects with a high load on language. Therefore, language difficulties have a high impact in the schooling of these children, as shown in previous studies carried out in English academic systems, such as the United Kingdom (Snowling et al., 2001; Conti-Ramsden et al., 2010), the United States (Hall and Tomblin, 1978), or Canada (Beitchman et al., 1996; Young et al., 2002).

Concerning curriculum adaptations, although these would be provided to improve the academic performance of children with DLD, only a quarter of the children received them. It seems that the most common measure was to make these children repeat a grade (57% of children). Besides, there was a statistical relationship between curriculum adaptations and grade retention, as all the children with curriculum adaptations also had repeated a course. However, there were also children from the DLD group with only grade retention (no curriculum adaptations applied), and without any of the two interventions (no curriculum adaptations, and no grade retention). Other studies have also shown a high percentage of grade retention in children with learning disabilities (Barnett et al., 1996) and it seems this might not be a good compensatory course of action (Owings and Kaplan, 2001), especially in cases of learning difficulties (Holmes and Matthews, 1984; Owings and Magliaro, 1998). In this sense, the present work shows the apparent inefficiency of these measures to compensate adequately for the language difficulties in most of the children with DLD, as shown by their academic results, which get worse at the end of primary education. Therefore, our data suggest that neither curriculum adaptations nor grade retention appeared to work adequately for these children in our educational context.

Regarding the second objective, we aimed to explore which processing skills measured at the beginning of schooling could best predict the academic results of these children at primary education. We collected these processing skills at age six, at the beginning of obligatory education in Spain, and before children learned to read. All processing skills that have been found delayed in children with DLD and/or other developmental disabilities (Buil-Legaz et al., 2016a) were considered as potential predictors of the average mark. Results confirmed partially our hypothesis, showing that rapid naming predicted the most variance of the academic outcomes, followed by phonological awareness, and, finally, comprehension of instructions when both groups were taken together. These three variables explained 75.8% of the variance in academic results on primary education. Nevertheless, only rapid naming accounted for a significant proportion of variance in the average mark in the DLD group, explaining 37.4% of the variance. Meanwhile, in the control group, phonological awareness accounted for 53.6% of the variance of the average mark.

A great body of research has found that rapid naming and phonological awareness are related with literacy in typical and atypical populations, and are considered as precursors of reading accuracy (Wolf et al., 2000; Gillon, 2004), but less is known about the relation between these processes and academic outcomes. In such a way, previous studies have shown that only children with DLD and low rapid automatized naming and phonological awareness abilities have also reading difficulties (Catts et al., 1999; Kelso et al., 2007). However, both abilities reflect different skills: rapid automatized naming bespeaks the fast retrieval of phonological codes from the lexicon (Claessen et al., 2013) and phonological awareness is considered as “the awareness of the sound structure, or phonological structure, of a spoken word” (Gillon, 2004). Therefore, these are complementary abilities, and while phonological awareness has been found highly related to decoding skills (Diamanti et al., 2017), rapid automatized naming has been related to spelling (orthographic) skills and processing speed (Wimmer, 1993; Kail and Hall, 1994; Manis et al., 1999; Stappen and Van Reybroeck, 2018), because rapid automatized naming involves rapid arbitrary associations between printed letters and sound (e.g., a figure and its shape, color, and size), whereas phonological awareness is more related to the learning of systematic spelling-sound correspondences.

Our results showed that rapid naming is the key factor for explaining academic outcomes in children with DLD. Meanwhile, phonological awareness had a higher impact on the academic outcomes of control children. Contrary to studies conducted in other languages, such as English (Snowling et al., 2001; Pentimonti et al., 2016), phonological awareness does not seem to impact the academic outcomes of children with DLD. This difference between studies conducted in different languages might arise as a result of the level of orthographic transparency of the language. In this way, the impaired phonological awareness ability of Spanish–Catalan children with DLD (see Buil-Legaz et al., 2016a) might be less important predicting academic outcomes because decoding is easier in transparent languages. Instead, the speeded and arbitrary orthographic associations that are specific to rapid naming were very important to explain academic results in children with DLD in a context in which more transparent languages are used. Thus, children with worse rapid naming skills would show a slowed cognitive processing that affect reading fluency and comprehension (Spring and Davis, 1988; Wimmer, 1993; Kail and Hall, 1994; Young and Greig Bowers, 1995), and would have more written orthographic errors (Manis et al., 1999), being both aspects crucial for academic achievements.

Other works have also found that rapid naming is the most important predictor of reading in transparent languages such as German, Dutch, or Italian in children with dyslexia and/or language impairment (Wimmer, 1993; de Jong and van der Leij, 2003; Brizzolara et al., 2006; Di Filippo et al., 2006; Vandewalle et al., 2010). Beyond the above-mentioned results, our study has related rapid naming with the academic outcomes, which would have a large influence on the future academic trajectory of children with DLD, leading to a higher risk of scholar failure (Young et al., 2002; Conti-Ramsden et al., 2010), which might in turn also lead to difficulties in employment opportunities at long-term (Carroll and Dockrell, 2010; Cleave et al., 2010; Durkin et al., 2012a,b). In this vein, Mulder et al. (2010) have also informed that processing speed seems to have an important role in explaining academic outcomes in premature children.

Nevertheless, the variance explained for the DLD group is lower than that of the control group, suggesting that other variables, different from processing skills, could be relevant to explain academic outcomes in children with DLD. Our results point that, in transparent languages, reading might not be as crucial as in other languages for school achievement in children with DLD. Therefore, further research exploring variables such as language profiles (e.g., vocabulary, morpho-syntax, phonological production, and discrimination), social context (e.g., educational implication of parents, level of education of parents) or literacy skills (e.g., letter naming and print concepts) would add new valuable pieces of information to this topic.

When both groups are taken together, another relevant predictor of academic outcomes is language comprehension (measured here by the comprehension of instructions test). Previous studies have also related this measure to reading, especially in reading comprehension (Buil-Legaz et al., 2016b). In this sense, Stage et al. (2003) showed that deficits in more than one language skill (comprehension and production) are at greater risk of experiencing reading difficulties and, consequently, academic difficulties. Nevertheless, in our context, this variable is only important to explain academic outcomes either considering both groups together or only the group of children with typical language development, maybe because all the children with DLD show a very low comprehension level. Finally, visual attention, that is highly related to reading (Gabrieli and Norton, 2012), is related to academic results only in typically developing children in the present study (see Table 3 for correlation values).

As discussed above, it seems that those variables that strongly predict academic achievement in children with and without DLD are related to spelling skills, the speed of processing, decoding in reading, and language comprehension. Therefore, those children who have difficulties with rapid naming, phonological awareness, and oral language comprehension would read very slowly, experience difficulties with spelling when they write, show decoding difficulties when they read, and exhibit oral and writing comprehension problems when they receive instructions. These abilities are fundamental for adequate learning in school and can lead to academic failure when disrupted, not only for children with DLD but also for all the children. Compensation measures must be applied as soon as these difficulties are detected, if possible, in the first years of schooling to prevent long-term academic difficulties. Moreover, these compensation measures should include the stimulation of these processing capacities, specially of rapid naming in children with DLD (Bus and van IJzendoorn, 1999; Al Otaiba et al., 2008; Duff and Clarke, 2011; Lanfranchi and Carretti, 2016; Stappen and Van Reybroeck, 2018), besides language stimulation (Duff and Clarke, 2011; Fricke et al., 2017), and the adequate measures of school adaptations (Owings and Kaplan, 2001; Kent, 2006) that include high-quality classroom environments, which have been linked to better decoding outcomes and, therefore, to academic achievements (Tambyraja et al., 2015).

Unfortunately, our study has not been able to analyze the type of curriculum adaptation measures applied to these children, as the official academic report did not include such information. However, the fact that there were no children with DLD with curriculum adaptations who did not repeat a course indicates that these measures were not working adequately. Therefore, more research is needed to disentangle the type of curriculum adaptations that these children received and to better understand the kind of school support that can help them overcome academic demands. Moreover, future studies would help to understand the causes of these academic outcomes, the reasons for grade retention, and would allow for further comparisons with other works including other languages and considering different academic systems.

Despite the relevance of the results obtained, one of the major limitations of our study is the relatively small number of participants. Therefore, results must be taken with some caution. This small number was due, in part, to the restrictive diagnostic inclusion criteria, and to the experimental mortality associated with longitudinal designs. These limitations are frequent in longitudinal studies due to difficulties in both the recruitment and maintenance of participants in the follow-up phases (see for example Snowling et al., 2001). Another potential limitation concerns the use of a syllable-level task to assess phonological awareness rather than a phoneme-level task, which might be more sensitive to detect differences between children with DLD and children in the control group. Future studies will benefit from the inclusion of a wider set of tasks devoted to assessing phonological awareness at different levels. Thus, further studies including a larger number of participants and extending the number of variables related to literacy (such as vocabulary, print concepts, and letter naming) shall help to add new valuable information on the relationship between processing skills and academic outcomes.

In brief, our study has several educational and clinical implications. First, we show that children with DLD experience academic difficulties during their primary education also in Spanish educational systems. Those children (with and without DLD) who had difficulties in rapid naming, phonological awareness, and oral language comprehension at the beginning of their schooling showed a higher probability of academic failure. Nevertheless, rapid naming was the key factor to explain academic outcomes in children with DLD, whereas phonological awareness was the most important predictor of academic outcomes in children without language difficulties. It is worthy of note that children with difficulties in both abilities are likely to have more spelling errors when they start writing, to experience difficulties in decoding when they start reading, and to have problems in comprehending oral and written commands/instructions. The disruption of these abilities, which are crucial to scholar learning, could turn into an academic failure; therefore, it is highly recommended that educational prevention measures consider the results of the present study in order to diminish academic failure, especially in children with language difficulties.

## Data Availability

The raw data supporting the conclusions of this manuscript will be made available by the authors, without undue reservation, to any qualified researcher.

## Author Contributions

EA-M designed the study, selected the children, analyzed the data, and wrote the first draft of the manuscript. LB-L administered and corrected the tests, tabulated the data, and corrected the paper. VS-A analyzed the data and corrected the manuscript. RL-P analyzed the data and corrected the manuscript. DA-R designed the study, analyzed the results, and wrote and corrected the manuscript.

## Conflict of Interest Statement

The authors declare that the research was conducted in the absence of any commercial or financial relationships that could be construed as a potential conflict of interest.

## Footnotes

1 http://www.uib.cat/recerca/estructures/comissions/cer/

## Abbreviations

DLD: developmental language disorder
SLI: specific language impairment
