# Supplementary material for: Academic Outcomes in Bilingual Children With Developmental Language Disorder: A Longitudinal Study
Source: Front Psychol. 2019 Mar 11;10:531. doi: 10.3389/fpsyg.2019.00531 (PMC6421289; doi:10.3389/fpsyg.2019.00531)
Supplement: Supplementary file 1 [file Table_1.pdf]

Appendix A: Summary of the NEPSY (Korkman et al., 1998) **tasks adapted to Spanish used to evaluate processing skills in DLD and control children.**

AREAS	MEASURE	TASK	EXAMPLE
ATTENTION	AUDITORY ATTENTION	Put a red square when the child hears "*Rojo*" (red).	*Negro, casa, pronto, así, escucha, ROJO, cuadrado, ahora, amarillo* (...)
	RESPONSE SET	Put a yellow square when the child hears "ROJO" (red), and a red square when hears "AMARILLO" (yellow), and a blue square when hears "AZUL" (blue).	*Allí, ahora, fino, negro, cuadrado, cosa, aburrido, AMARILLO, pon, ahora, AZUL (...)*
	VISUAL ATTENTION	Encircle all the faces that match two example ones on an A3 page with different faces.	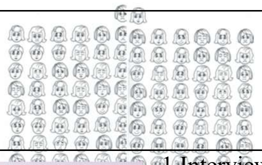
PHONOLOGICAL PROCESSING	PHONOLOGICAL AWARENESS	Recognize a word without a part of them	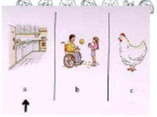 1.Interviewer: *cocina* (kitchen), *niños*(children), *gallina* (hen) 2. Interviewer: *cina* (chen) 3. Child: say sounds or point to the picture
VERBAL SHORT-TERM MEMORY	SENTENCE REPETITION	Sentence repetition of increased difficulty	1. "*Duerme bien*" (sleep well) (...) 17. "*El próximo miércoles a las dos de la tarde nuestro equipo de fútbol jugará un partido en un campeonato que se celebrará en el estadio.*" (Next Friday at two o'clock our football team will play a match in a championship to be held at the stadium)
	NARRATIVE MEMORY	Remember details from a history	The interviewer read a history: *Juan era un niño cuyo mejor amigo era Sultán …* (John was a child whose better friend was Sultán….) The child must explain the history. If some details are not referred, an induced remember was done through questions like *What is the name of the child?*
ACCESS TO LANGUAGE	RAPID NAMING	Quickly say the form, color, and size	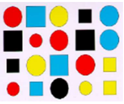 1. Child: Red circle big Square blue big …
	VERBAL FLUENCY	Build word in semantic categories	Words of animals Words of foods
LANGUAGE COMPREHENSION	COMPREHENSION OF INSTRUCTIONS	Point to the image that corresponds to the given instruction.	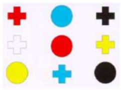 Example 1. The blue cross and the yellow cross
